# Diosgenin Inhibits ROS Generation by Modulating NOX4 and Mitochondrial Respiratory Chain and Suppresses Apoptosis in Diabetic Nephropathy

**DOI:** 10.3390/nu15092164

**Published:** 2023-04-30

**Authors:** Yujie Zhong, Lei Wang, Ruyi Jin, Jiayu Liu, Ruilin Luo, Yinghan Zhang, Lin Zhu, Xiaoli Peng

**Affiliations:** 1 College of Food Science and Engineering, Northwest A&F University, Xianyang 712100, China; 2 Qinling National Botanical Garden, Xi’an 710061, China

**Keywords:** diosgenin, diabetic nephropathy, NOX4, mitochondria, apoptosis, ER stress

## Abstract

Diosgenin (DIO) is a dietary steroid sapogenin possessing multiple biological functions, such as the amelioration of diabetes. However, the remission effect of DIO on diabetic nephropathy (DN) underlying oxidative stress and cell apoptosis remains unclear. Here, the effect of DIO on ROS generation and its induced cell apoptosis was studied in vitro and in vivo. Renal proximal tubular epithelial (HK-2) cells were treated with DIO (1, 2, 4 µM) under high glucose (HG, 30 mM) conditions. DN rats were induced by a high-fat diet combined with streptozotocin, followed by administration of DIO for 8 weeks. Our data suggested that DIO relieved the decline of HK-2 cell viability and renal pathological damage in DN rats. DIO also relieved ROS (O_2_^−^ and H_2_O_2_) production. Mechanistically, DIO inhibited the expression of NOX4 and restored mitochondrial respiratory chain (MRC) complex I-V expressions. Further, DIO inhibited mitochondrial apoptosis by ameliorating mitochondrial membrane potential (MtMP) and down-regulating the expressions of CytC, Apaf-1, caspase 3, and caspase 9, while up-regulating Bcl2 expression. Moreover, the ER stress and its associated cell apoptosis were inhibited through decreasing PERK, *p*-PERK, ATF4, IRE1, *p*-CHOP, and caspase 12 expressions. Collectively, DIO inhibited ROS production by modulating NOX4 and MRC complexes, which then suppressed apoptosis regulated by mitochondria and ER stress, thereby attenuating DN.

## 1. Introduction

Diabetes mellitus (DM) is one of the common chronic metabolic diseases with an increased incidence in recent years. Globally, approximately 451 million adults (20–79 years old) suffer from DM in 2017, and the number of people with diabetes is estimated to reach 700.2 million by 2045 [1]. Persistent hyperglycemia can lead to serious micro- and macro-vascular complications [2]. Diabetic nephropathy (DN) is a serious microvascular complication that occurs in the condition of hyperglycemia, affecting about 1/3–2/5 of patients with diabetes [3], and has become the first factor leading to end-stage renal disease [4].

Oxidative stress is highly positively correlated with the progression of DN [5,6]. The over-production of ROS that exceeds the limit of antioxidant capacity will cause oxidative stress, which then oxidizes various elements including carbohydrates, lipids, proteins, and DNA. In hyperglycemic conditions, divergent sources operate in the generation of ROS, such as NADH/NADPH oxidase, the mitochondrial respiratory chain (MRC), NO synthase, and xanthine oxidase [7,8]. MRC is the main site generating ROS which produces excessive electron leakage in MRC complex I and in the interface between MRC complex III and coenzyme Q [9]. NADPH oxidase is another site producing ROS which consists of 7 members, including NOXs1-5, DUOX1, and DUOX2 [10]. Among these members, NOX4 is the highest expressed isoform mainly generating H_2_O_2_ under renal pathological conditions, such as chronic kidney diseases and DN [11]. Inhibition of NOX4 could relieve DN. For example, Liang et al. [12] indicated that Salvianolate and NOX1/NOX4 inhibitor-GKT137831 prevented high glucose (HG)-induced oxidative podocyte damage by modulating NOX4 activity. Yang et al. [13] showed that Huidouba inhibited HG-induced oxidative stress and apoptosis in podocytes through down-regulating NOX4 expression.

Under hyperglycemia, severe oxidative stress leads to sustained damage to mtDNA [14], which in turn promotes the generation of ROS, followed by intense cell death via necrosis or apoptosis [15,16]. Emerging evidence showed that oxidative stress, mtDNA damage, mitochondrial dysfunction, and cell apoptosis were observed in HK-2 cells, podocytes, and mesangial cells in the case of HG [17,18]. In addition, endoplasmic reticulum (ER) stress can also induce apoptosis, which then accelerates DN progression [19]. ER is an organelle responsible for protein processing. The increase in endogenous ROS, the dysregulation of cellular redox, and the accumulation of misfolded proteins under hyperglycemia conditions are the main factors that trigger ER stress [20]. Studies have suggested that the inhibition of oxidative stress by *N*-acetylcysteine, tempol, ramipril (pharmacological agents to inhibit NADPH oxidase), and GKT137831/GKT136901 (NOX4 inhibitors) significantly relieved DN by suppressing ER stress and its associated cell apoptosis [21,22,23,24].

Diosgenin (DIO), belonging to the category of steroidal sapogenin, is primarily obtained from wild yam (*Dioscorea villosa*), fenugreek, *smilax bockii* warb, and *dioscorea nipponoca* makino. It has excellent pharmacological activity in the alleviation of hyperlipidemia, diabetes, diabetic kidney injury, and diabetic liver injury [25,26,27,28,29]. For example, DIO at 22.1 and 44.2 mg/kg/d exerted hypoglycemic and anti-oxidant enzyme-enhancing effects in SD rats induced by a high-fat diet (HFD) [28]. Since DIO has an excellent ability to enhance anti-oxidant enzyme activity and then prevents oxidative stress, we wonder whether DIO could relieve DN through regulating ROS generation sites (MRC and NOX4), ROS targets (mitochondria and ER), and the ROS target-mediated cell apoptosis. In our study, the alleviative effect of DIO on DN was investigated, focusing on MRC, NOX4, and apoptosis regulated by mitochondria and ER stress.

## 2. Methods

### 2.1. Cell Culture and Treatment

Renal proximal tubular epithelial (HK-2) cells were provided by Purosa life technology (Wuhan, Hubei, China). The HK-2 cells were cultured in DMEM media of 5.5 mM glucose (Gibco, Carlsbad, CA, USA) added with fetal bovine serum (10%, Gibco) and penicillin-streptomycin solution (1%, Beyotime, Shanghai, China) at an incubator (37 °C, 5% CO_2_). When they were cultured to 70–80% fusion, the cells were treated with different concentrations of HG (10, 20, 30, 40, and 50 mM) for 24 h, 30 mM HG for different durations (6, 12, 24, and 48 h), and DIO (1, 2, and 4 µM) for 24 h under HG (30 mM) condition. Mannitol (30 mM) was used as hypertonic control.

### 2.2. Cell Viability

The cell culture and treatment refer to Section 2.1. After 24 h treatment with HG and DIO, the 10 µL MTT solution (Sigma, St. Louis, MO, USA) of 5 mg/mL was added to 100 µL cell fluid. After incubation (37 °C, 4 h), the fluid in each well was sucked out and then 150 µL DMSO was added. The cell absorbance at 570 nm measured by the multimode reader (Spark, Tecan Austria GmbH, Grödig, Austria) indicates the cell viability.

### 2.3. DHE Fluorescence/O_2_^−^ Analysis

Dihydroethidium (DHE, Beyotime) fluorescent probe was used to measure O_2_^−^. The cells were cultured and treated according to Section 2.1. After treatment with HG and DIO for 24 h, the HK-2 cells were then stained with DHE reagent (Beyotime) (37 °C, 30 min) in dark. After cleaning the DHE solution on the cell surface with PBS, the visualization and photography were conducted under the inverted fluorescence microscope (Lecia DMI8, Wetzlar, Germany).

### 2.4. H_2_O_2_ Analysis

The Hydrogen Peroxide Assay Kit (Beyotime) was used to detect the H_2_O_2_ level in HK-2 cells. The HK-2 cells (approximately 2 × 10^6^) were homogenized with lysate (200 µL) in an icy bath. Next, the obtained homogenate was centrifugated (11,000× *g*, 4 °C for 5 min) to collect the supernatant fluid. Followed by, the supernatant was reacted with the test solution provided by the kit at 25 °C for 30 min. Finally, the absorbance at 560 nm of the samples was detected by the multifunctional microplate reader (Spark).

### 2.5. DAPI Staining

The cell culture and treatment refer to Section 2.1. After 24 h treatment with DIO and HG, the cells were stained with 10 μg/mL DAPI staining solution (Beyotime) at dark for 5 min. Next, the cells experience three washes with PBS for 3 min each. Finally, visualization and photography were performed with an inverted fluorescence microscope (Lecia DMI8).

### 2.6. Immunofluorescence Assay

The expression of NOX4 in HK-2 cells was measured by immunofluorescence, as described by our previous study [30]. In brief, the HK-2 cells were blocked with goat serum (37 °C, 20 min), incubated with NOX4 antibody (Proteintech, 14347-1-AP) at 4 °C for 12 h, and then hatched with secondary anti-rabbit antibody labeled by Alexa fluor 488 (Beyotime) for 30 min in the dark. Then the nucleus of cell was stained with DAPI solution (Beyotime). The images of NOX4 immunofluorescence were visualized and photographed using an inverted fluorescence microscope (Lecia DMI8).

### 2.7. Mitochondrial Membrane Potential (MtMP) Measurement

The MtMP of the cells was measured by the JC-1 probe (Beyotime). Briefly, the HK-2 cells were cultured and treated as Section 2.1. After 24 h treatment with DIO and HG, the cells were incubated with the JC-1 probe for 20 min at 37 °C, followed by two washes with dyeing buffer (1×). The JC-1 fluorescence images were visualized and photographed using an inverted fluorescence microscope (Lecia DMI8).

### 2.8. Animals and Treatments

The special pathogen-free Sprague-Dawley (SD) male rats (200 ± 20 g) were provided by Chengdu Dossy Experimental Animals Co., Ltd (Sichuan, Chengdu, China). All rats were housed under a controlled condition of 20 ± 2 °C of temperature, 60–70% of humidity, and 12 h light/12 h dark cycle. After 1 week of adaptive feeding, the rats were randomly divided into the control group (fed with standard chow) and the DN group. The model construction refers to our previous description [30]. Rats with random blood glucose > 16.7 mM were considered as DM and then the rats were divided into 10 mg/kg bw of DIO application group, 20 mg/kg bw of DIO application group, 8 rats in every group. DIO was given to rats for 8 weeks, orally and daily. All animal procedures were approved by the Animal Ethics Committee of Northwest Agriculture and Forestry University (N20071065).

### 2.9. Histological Analysis

The periodic acid and Schiff’s (PAS), hematoxylin and eosin (H&E), and Masson trichromatic dye analyses were processed according to our previous study [30]. Then the changes in kidney structure were visualized and photographed using a stereomicroscope (NikonSMZ25, Tokyo, Japan).

### 2.10. Western Blot Assay

The protein expressions of the kidneys and cells were detected by Western blot as described previously [31,32]. The primary antibodies, including NOX4 (14347-1-AP), Bcl2 (12789-1-AP), NDUFA4 (16480-1-AP), Apaf-1 (21710-1-AP), SDHA (14865-1-AP), UQCRC2 (14742-1-AP), Bax (50599-2-lg), COX17 (11464-1-AP), CytC (10993-1-AP), caspase 3 (19677-1-AP), IRE1 (27528-1-AP), caspase 9 (10380-1-AP), and ATF4 (10835-1-AP) were purchased from Proteintech. The primary antibodies: *p*-CHOP (bs-5177R), PERK (bs-2469R), Caspase 12 (bs-1105R), and *p*-PERK (bs-3330R) were purchased from Bioss. The ATP6 primary antibody (AF6261) and the HRP-labeled secondary antibody (A0208) were purchased from Beyotime.

### 2.11. Immunohistochemistry Assay

The immunohistochemistry was performed as described by our previous study [30]. The ATP8 primary antibody (ab130441) was purchased from Abcam. The primary antibodies including Bax (50599-2-lg), CytC (10993-1-AP), Bcl2 (12789-1-AP), and caspase 3 (19677-1-AP) were provided by Proteintech. The *p*-PERK (bs-3330R) primary antibody was purchased from Bioss.

### 2.12. Statistical Analysis

All data were presented as the mean ± standard deviation (SD). Significant differences between groups were analyzed by one-way ANOVA, followed by Duncan’s test for multiple comparisons using SPSS 20.0. A *p* value less than 0.05 was considered as statistical significance.

## 3. Results

### 3.1. DIO Relieved the Decline of Cell Viability and ROS Production in HK-2 Cells

**Figure 1:**
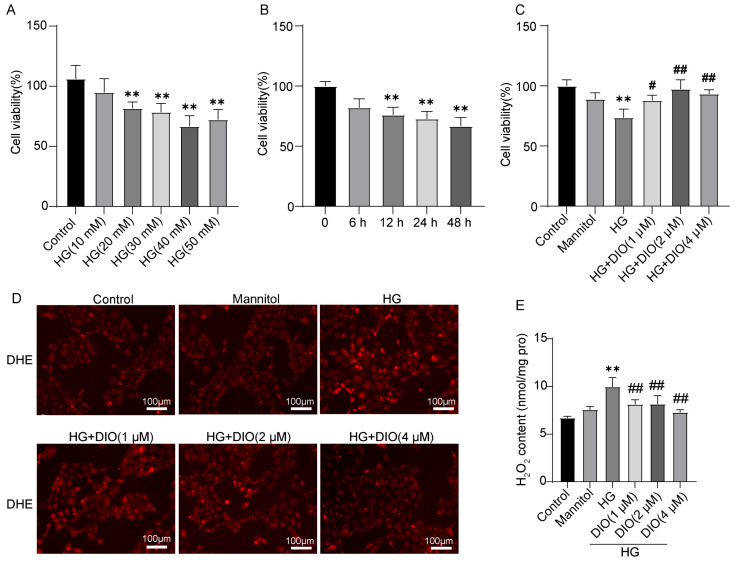
DIO relieved the decline of cell viability and the production of ROS in HK-2 cells. The HK-2 cell viability after treatment with (**A**) different concentrations of HG (10, 20, 30, 40, and 50 mM) treatment for 24 h and (**B**) 30 mM HG treatment for different time (6, 12, 24, and 48 h). (**C**) HK-2 cell viability after treatment with DIO (1, 2, and 4 µM) and HG (30 mM). (**D**) The representative DHE staining images, ×200, scale bars = 100 µm. (**E**) H_2_O_2_ content. Data were expressed as mean ± SD (*n* = 5). ** *p* < 0.01 indicate versus control group; ^#^ *p* < 0.05 and ^##^ *p* < 0.01 indicate versus HG group.

To detect the protective effect of DIO against oxidative stress, the cell viability and ROS contents were detected. In this study, the HK-2 cell viability decreased significantly after different concentrations of HG (20, 30, 40, and 50 mM) treatment for 24 h and 30 mM HG treatment for 6, 12, 24, and 48 h (Figure 1A,B). DIO (1, 2, and 4 µM) supplementation restored the cell viability in a dose-dependent manner (Figure 1C). O_2_^−^ and H_2_O_2_ are the two main ROS. The fluorescence intensity of DHE is positively correlated with the content of O_2_^−^. The results of DHE staining showed that HG treatment resulted in a significant increase of O_2_^−^ content, while it was reversed in the DIO-treated groups (Figure 1D). Additionally, DIO relieved the increase of H_2_O_2_ content in HG-treated HK-2 cells (Figure 1E). These results indicated that DIO increased cell viability and reduced ROS levels in HK-2 cells.

### 3.2. DIO Inhibited ROS Production by Regulating MRC Complexes and NOX4 in HK-2 Cells

**Figure 2:**
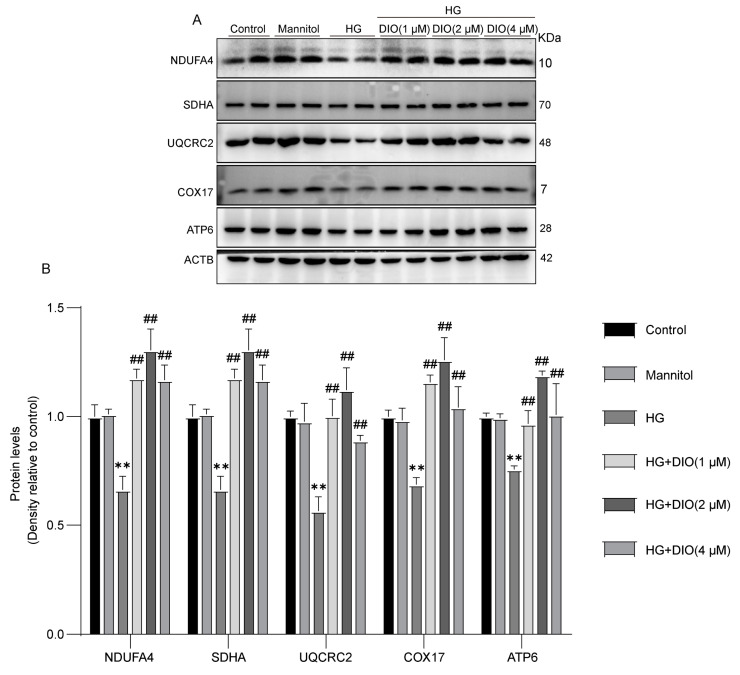
DIO restored MRC complexes I-V expressions in HK-2 cells. (**A**) The representative immunoblotting images of MRC complexes I-V and ACTB. (**B**) Quantitative analyses of MRC complexes I-V protein expressions. Data were expressed as mean ± SD (*n* = 2). ** *p* < 0.01 indicate versus control group; and ^##^ *p* < 0.01 indicate versus HG group.

Since the MRC is the primary site generating ROS, the protein levels of MRC complexes are important indicators to assess ROS production. In this study, the expressions of mitochondrial complex I-V (NDUFA4, SDHA, UQCRC2, COX17, and ATP6) were significantly inhibited in HG-treated HK-2 cells, while these alterations were ameliorated in the DIO-treated groups (Figure 2A,B and Figure S6). Therefore, it can be concluded that DIO improved the disorder of MRC.

**Figure 3:**
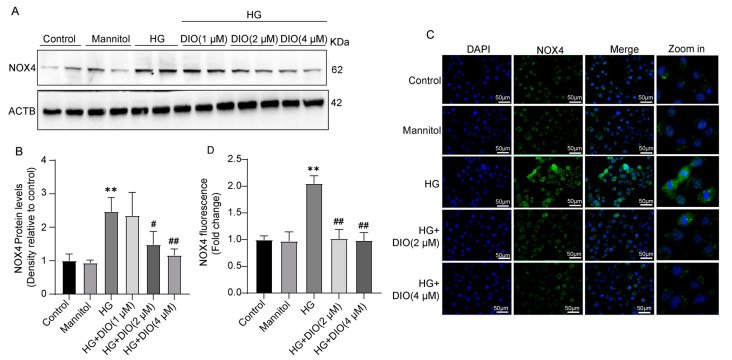
DIO suppressed the expression of NOX4 in HK-2 cells. (**A**) The representative immunoblotting images of NOX4 and ACTB. (**B**) Quantitative analysis of NOX4 protein expression. (**C**) The representative immunofluorescence staining images of NOX4, ×400, scale bars = 50 µm. (**D**) Quantitative analysis of NOX4 immunofluorescence. Data in (**A**,**B**) (*n* = 2) and in (**C**,**D**) (*n* = 5) were presented as mean ± SD. ** *p* < 0.01 indicate versus control group; ^#^ *p* < 0.05 and ^##^ *p* < 0.01 indicate versus HG group.

NADPH oxidase is another main souse of ROS. In the kidneys, NOX4 is the most critical subunit of NADPH oxidase with the highest level, which is responsible for H_2_O_2_ production. In this study, the NOX4 expression in HK-2 cells were detected by both Western blot and immunofluorescence. The data suggested that NOX4 expression was significantly up-regulated in HK-2 cells treated with HG and it was effectively inhibited by the co-treatment of DIO (Figure 3 and Figure S6). Therefore, it can be concluded that DIO suppressed ROS production by inhibiting the NOX4 expression in the kidneys.

### 3.3. DIO Improved MRC Disorder and Inhibited NOX4 Expression in DN Rats

**Figure 4:**
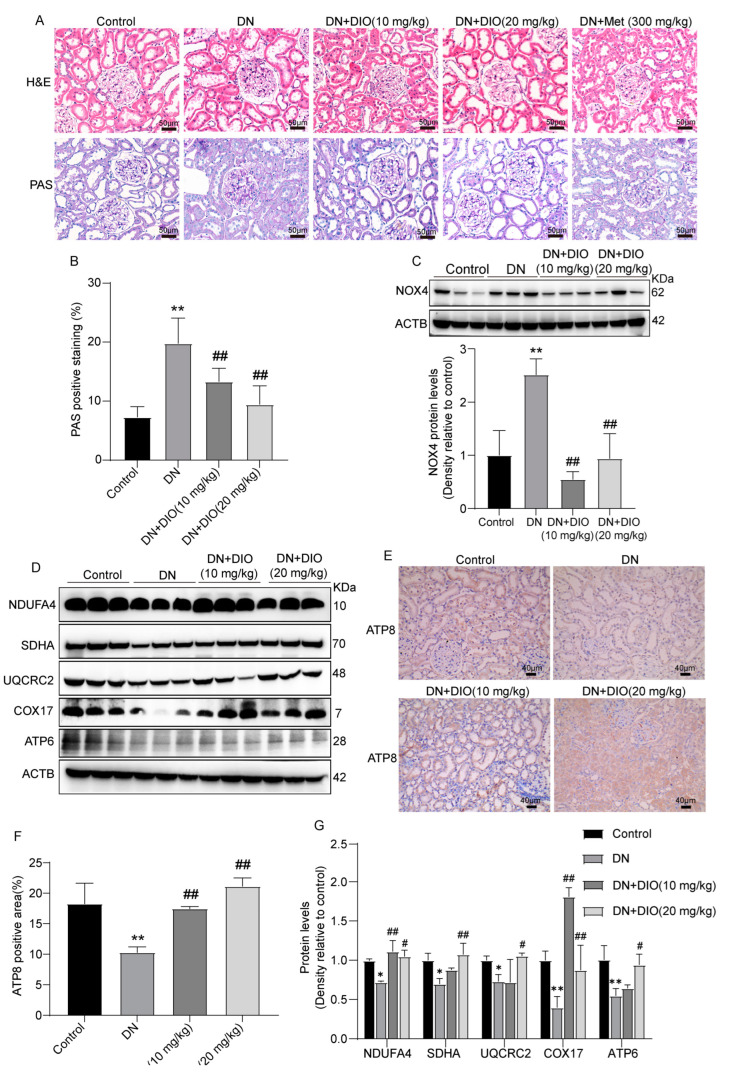
DIO restored MRC complex expressions and inhibited the expression of NOX4 in the rat kidney tissues. (**A**) The representative H&E and PAS staining images of rat kidneys, ×200, scale bars = 50 µm. (**B**) Quantitative analysis of PAS positive staining. (**C**) The representative immunoblotting image and quantitative analysis of NOX4 protein expression. (**D**) The representative immunoblotting images of MRC complexes I-V and ACTB. (**E**) The representative immunohistochemistry images of ATP8, ×200, scale bars = 40 µm. (**F**) Quantitative analysis of ATP8 immunohistochemistry. (**G**) Quantitative analyses of MRC complexes I–V protein expressions. Data were expressed as mean ± SD (*n* = 3). * *p* < 0.05 and ** *p* < 0.01 indicate versus control group; ^#^ *p* < 0.05 and ^##^ *p* < 0.01 indicate versus HG group.

To further detect the protective effect of DIO against DN, in vivo study was conducted in SD rats. Firstly, DIO relieved the kidney damage of DN rats. H&E and PAS staining showed that DN rats had significant kidney histological changes including mesangial matrix deposition, glomerular hypertrophy, and glomerular and tubular basement membrane thickening. The application of DIO (10 and 20 mg/kg) significantly relieved the above pathological changes (Figure 4A,B). In addition, the levels of urine protein, serum urea nitrogen, and serum creatinine were reduced by DIO (Figure S1). Next, the expressions of NOX4 and MRC complexes were detected in rats. Consistent with the data obtained in vitro, DIO relieved the up-regulation of NOX4 in the kidneys of rats (Figure 4C and Figure S6). DIO also restored the protein expressions of MRC complexes I-V (Figure 4D–G and Figure S6). These data showed that DIO ameliorated diabetic kidney damage, suppressed NOX4 expression, and improved the expressions of complex I–V of MRC in DN rat kidneys.

### 3.4. DIO Attenuated Mitochondrial Dysfunction and Its-Mediated Cell Apoptosis in HK-2 Cells

**Figure 5:**
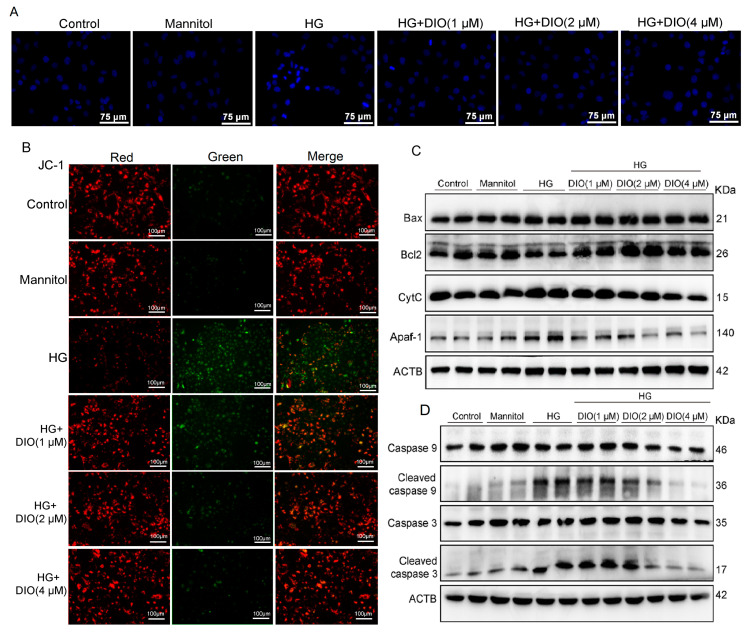
DIO relieved mitochondrial dysfunction and its-mediated HK-2 cell apoptosis. The representative (**A**) DAPI staining images (×400, scale bars = 75 µm) and (**B**) JC-1 staining images (the measurement of MtMP, ×200, scale bars = 100 µm). (**C**) The representative immunoblotting images of proteins in the mitochondrial apoptosis pathway (Bax, Bcl2, CytC, and Apaf-1) and ACTB. (**D**) The representative immunoblotting images of caspase 9, caspase 3, and ACTB. Data in (**A**,**B**) (*n* = 5) and in (**C**,**D**) (*n* = 2) were presented as mean ± SD.

Oxidative stress can cause severe damage to mtDNA and then cause mitochondria-mediated cell apoptosis. DAPI is common staining to detect cell apoptosis. The DAPI staining showed that HG exposure caused HK-2 cell apoptosis (dense or fragmented dense hyperchromatic nucleus) and DIO relieved the cell apoptosis (Figure 5A). The decline of MtMP is a marker of early cell apoptosis and the JC-1 probe can indicate the changes in MtMP. In this study, the data of JC-1 staining showed that red/green fluorescence intensity decreased greatly, suggesting that the MtMP decreased greatly in HG-treated HK-2 cells, while, DIO co-treatment improved the MtMP significantly (Figure 5B). Moreover, treatment with HG in HK-2 cells resulted in the up-regulation of mitochondrial pro-apoptotic proteins (Apaf-1, CytC, cleaved caspase 3, and cleaved caspase 9) and the down-regulation of anti-apoptotic protein-Bcl2. Conversely, DIO treatment down-regulated the expressions of the above pro-apoptotic proteins, while up-regulated Bcl2 expression, an anti-apoptotic protein (Figure 5C,D, Figures S2 and S6). Therefore, we summarized that DIO relieved mitochondrial disorder and its-mediated cell apoptosis in HK-2 cells.

### 3.5. DIO Ameliorated Mitochondria-Mediated Cell Apoptosis in DN Rats

**Figure 6:**
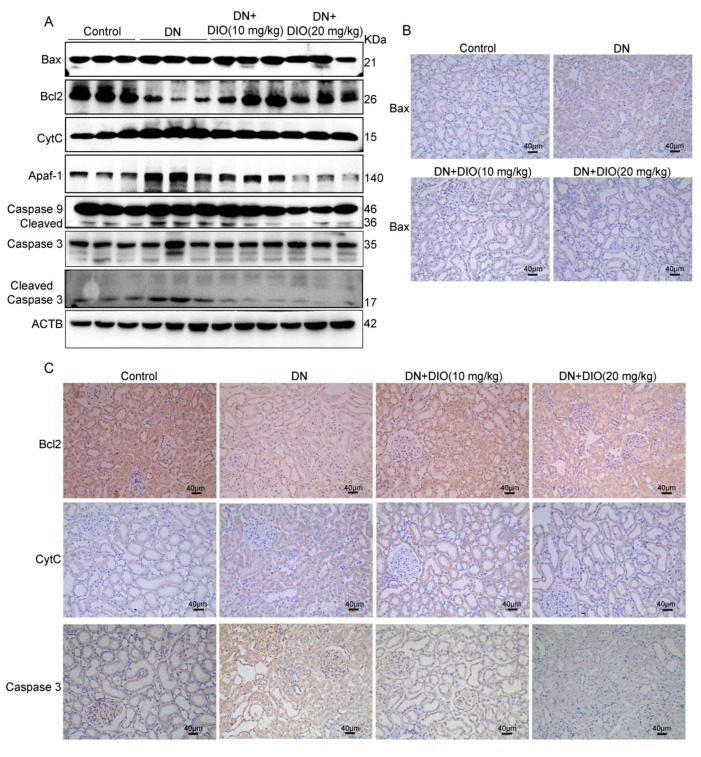
DIO ameliorated mitochondrial dysfunction and apoptosis in the kidney of diabetic rats. (**A**) The representative immunoblotting images of ACTB and proteins in the mitochondrial apoptosis pathway (Bax, Bcl2, CytC, Apaf-1, caspase 9, and caspase 3). The representative immunohistochemistry images of (**B**) Bax (×200, scale bars = 40 µm) and (**C**) Bcl2, CytC, and Caspase 3 (×200, scale bars = 40 µm). Data were expressed as mean ± SD (*n* = 3).

To further determine whether DIO ameliorates mitochondria-mediated cell apoptosis in vivo, the expressions of mitochondrial apoptotic proteins in rat kidney tissues were measured by Western blot and immunohistochemistry. Our results indicated that the up-regulation of mitochondrial pro-apoptotic proteins (Bax, CytC, Apaf-1, cleaved caspase 3, and cleaved caspase 9) were inhibited by DIO. While, the down-expression of Bcl2, an anti-apoptotic protein, was restored by DIO application (Figure 6, Figures S3, S6 and S7). Therefore, DIO protected against cell apoptosis by suppressing the mitochondrial apoptotic pathway in DN rats.

### 3.6. DIO Ameliorated ER Stress and Its-Mediated Cell Apoptosis in HK-2 Cells and DN Rats

**Figure 7:**
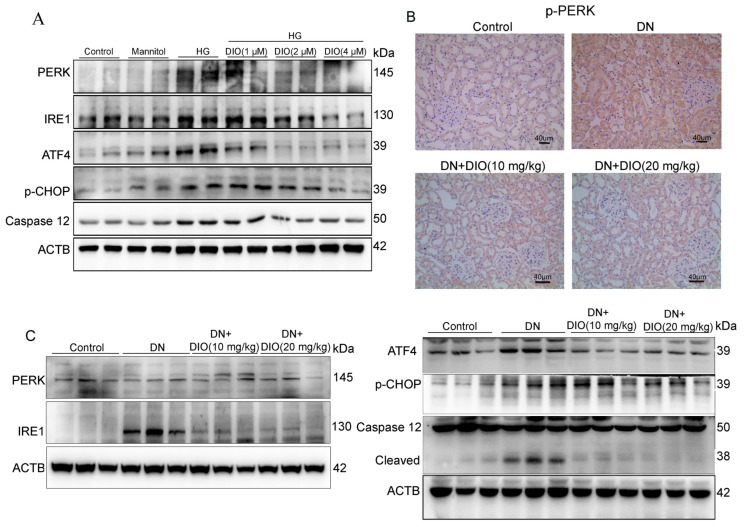
DIO alleviated ER stress and its-mediated cell apoptosis in HK-2 cells and DN rats. (**A**) The representative immunoblotting images of ACTB, PERK, IRE1, ATF4, Caspase 12, and *p*-CHOP in HK-2 cells. (**B**) The representative immunohistochemistry images of *p*-PERK in rat kidneys, ×200, scale bars = 40 µm. (**C**) The representative immunoblotting images of ACTB, PERK, IRE1, Caspase 12, ATF4, and *p*-CHOP in the kidneys of DN rats. Data in A (*n* = 2) and in B-C (*n* = 3) were expressed as mean ± SD.

Oxidative stress can also induce ER stress and its-mediated cell apoptosis, which also contributes to the occurrence and development of DN. The ER stress associated apoptosis is regulated by transmembrane proteins (IRE1 and PERK) and their downstream proteins including CHOP, ATF4, and caspase 12. Thus, these protein expressions were detected by Western blot and immunohistochemistry in this study. As shown in Figure 7A and Figure S4, the expressions of ER stress-related proteins (IRE1, ATF4, PERK, *p*-CHOP, and Caspase 12) were up-regulated significantly in HK-2 cells treated with HG. DIO inhibited the up-regulation of these proteins. Consistent with the results obtained in vitro, the up-expressions of IRE1, *p*-PERK, ATF4, *p*-CHOP, and caspase 12 were relieved by DIO in vivo (Figure 7B,C and Figures S5–S7). Altogether, the above data showed that DIO suppressed cell apoptosis by inhibiting ER stress in HK-2 cells and DN rats.

## 4. Discussion

Oxidative stress refers to the imbalance between the oxidative and anti-oxidant systems in the organism, making it prone to oxidation [33]. Numerous studies have suggested that oxidative stress is involved in the occurrence of DN. In the condition of hyperglycemia, oxidative stress can lead to changes in renal metabolism and hemodynamics, causing renal vascular damage-DN [34].

The current research focus is to develop natural antioxidants to prevent DN, such as vitamin C, vitamin E, vitamin D, resveratrol, quercetin, coenzyme Q10, curcumin, green tea extract, guava extract, etc. [35]. DIO is a natural steroidal sapogenin possessing excellent anti-oxidant activity and has a potent intervention effect on diabetes and its complications. Pharmacological studies have shown that DIO could alleviate the complication of DM by inhibiting oxidative stress. For example, DIO intervention for 5 weeks alleviated the cognitive impairment of STZ-induced diabetic rats by inhibiting the expressions of inflammatory cytokines and enhancing the activities of anti-oxidant enzymes [36]. DIO administration for 28 days can prevent STZ-induced kidney injury by preventing inflammatory and oxidative stress [37]. However, the specific mechanism by which DIO alleviates oxidative stress in DN has not been cleared yet. Therefore, our study aims to investigate the ameliorative effect of DIO against DN in terms of ROS sources (MRC and NOX4) and ROS targets (mitochondrial and ER stress) in HK-2 cells and SD rats. 

In the present study, DIO relieved the decrease in HK-2 cell viability after HG treatment. Next, the anti-oxidant activity of DIO was investigated. The excessive production of O_2_^−^ and H_2_O_2_ was observed in HK-2 cells exposed to HG. As expected, DIO significantly decreased O_2_^−^ and H_2_O_2_ levels. Studies showed that ROS generated by the disordered NADPH oxidase and MRC under HG condition contributes to the progression of DN. In the condition of hyperglycemia, damaged mitochondria produce excessive electron leakage in MRC complex I and in the interface between MRC complex III and coenzyme Q [38]. Studies have shown that the activities of MRC complexes I and III were reduced significantly in the kidney of *db*/*db* mice and podocytes treated with HG. And the enhancement of MRC complex I expression can inhibit the production of ROS [39,40]. In this study, the down-regulations of MRC complexes I-V occurred in HK-2 cells exposed to HG and in DN rat kidneys. While, DIO co-treatment restored the expressions of MRC complex-related proteins, which indicates that DIO could reduce ROS levels via regulating MRC. NOX4 is the most important isoform of NADPH oxidase, which is mainly responsible for the generation of H_2_O_2_ in the kidneys [41]. The increased NOX4 expression in HG-treated tubular epithelial cells, mesangial cells, podocytes, and DN rat/mice kidneys were observed [11,12,42,43]. Thus, NOX4 is considered a potential target for the remission of DN. For example, Yao et al. suggested that the inhibition of NOX4 can alleviate HK-2 cell apoptosis by regulating the Notch pathway [44]. In this study, NOX4 expression was up-regulated in HK-2 cells under HG conditions. DIO significantly inhibited its expression. Besides, the up-regulated NOX4 in the kidneys of DN rats was inhibited by DIO, suggesting that DIO can inhibit ROS production by down-regulating NOX4 expression.

The kidneys require massive mitochondria to provide energy, ensuring normal structure and function. The over-produced ROS derived from the defective MRC could in turn impair mitochondrial function [45]. Oxidative damage to mtDNA can further induce ROS production, forming a vicious cycle that ultimately leads to cell death [16]. Mitochondria are positioned as the “guards” of apoptosis [46]. Mitochondria-mediated cell apoptosis is characterized by the collapse of MtMP and the disorder of Bax/Bcl2 ratio. Then CytC was released from mitochondria to the cytoplasm. Finally, an oligomeric apoptosome was formed and caspase 9 was activated [47,48,49]. Inhibition of mitochondria-mediated apoptosis can alleviate DN. For example, administration with Hydroxysafflor yellow A for 8 weeks improved renal injury in type II diabetic rats by suppressing pro-apoptotic protein-Bax and apoptosis executor-Caspase 3 expressions, but enhancing anti-apoptotic protein-Bcl2 expression [50]. In our study, DIO relieved the dense and fragmented dense hyperchromatic nucleus, enhanced MtMP (a sign of early apoptosis), and decreased the expressions of CytC, Bax, Apaf-1, cleaved caspase 3, and cleaved caspase 9 in HG-treated HK-2 cells. Additionally, the Bcl2 expression was up-regulated. In addition, the up-regulations of CytC, Bax, Apaf-1, cleaved caspase 3, and cleaved caspase 9 were relieved, while the down-regulation of Bcl2 was restored in the kidneys of DN rats.

In addition to mitochondria, ER stress can also cause cell apoptosis [51]. Under normal physiological conditions, ER stress is an adaptive response to cells, which degrades misfolded or unfolded proteins [52]. However, prolonged ER stress can result in cell apoptosis through activating ATF4, CHOP, and Caspase 12, when they receive signals from the transmembrane proteins of PERK and IRE1 [53]. The prevention of ER stress and its associated cell apoptosis is a potential target for DN treatment. Studies suggested that ER stress inhibitors, including Tauroursodeoxycholic acid and 4-phenyl-butyric acid, could alleviate DN by inhibiting ER stress and its associated cell apoptosis [54,55]. Moreover, there is a crosstalk between ER stress and oxidative stress in the development of DN. Many substances with anti-oxidant capacity can also inhibit ER stress and associated apoptosis, such as Resveratrol [56], Tanshinone IIA [57], and epigallocatechin gallate [58]. In this study, except for the anti-oxidant capacity, DIO also suppressed ER stress and its associated cell apoptosis by preventing the expressions of PERK, *p*-PERK, *p*-CHOP, ATF4, IRE1, and caspase 12.

## 5. Conclusions

Collectively, our study indicated that DIO protected against diabetic kidney damage by inhibiting ROS production and cell apoptosis in vitro and in vivo. DIO inhibited ROS production mainly through down-regulating NOX4 expression and up-regulating the expressions of MRC complex I–V. Subsequently, the cell apoptosis regulated by mitochondria and ER stress was suppressed by DIO.

## Data Availability

The data presented in this study are available on request from the corresponding author.

## Supplementary Materials

The following supporting information can be downloaded at: https://www.mdpi.com/article/10.3390/nu15092164/s1, Figure S1: DIO reduced urine protein, serum urea nitrogen, and serum creatinine levels in DN rats; Figure S2: DIO ameliorated mitochondria-mediated apoptosis in HG-treated HK-2 cells; Figure S3: DIO ameliorated mitochondria-mediated apoptosis in the kidney of DN rats; Figure S4: DIO ameliorated ER stress-mediated apoptosis in HK-2 cells; Figure S5: DIO ameliorated ER stress-mediated apoptosis in the kidneys of DN rats; Figure S6: Western blot images and their corresponding internal controls; Figure S7: The representative immunohistochemistry images and their partial enlarged images of Bax. Bcl2, CytC, Caspase 3, ATP8, and p-PERK.
