# Supplementary material for: Diosgenin Inhibits ROS Generation by Modulating NOX4 and Mitochondrial Respiratory Chain and Suppresses Apoptosis in Diabetic Nephropathy
Source: Nutrients. 2023 Apr 30;15(9):2164. doi: 10.3390/nu15092164 (PMC10181383; doi:10.3390/nu15092164)
Supplement: Supplementary file 1 [file nutrients-15-02164-s001.zip › nutrients-2346259-supplementary.pdf]

Supplementary materials

Diosgenin inhibits ROS generation by modulating NOX4 and mitochondrial respiratory chain and suppresses apoptosis in diabetic nephropathy

Yujie Zhong[1], Lei Wang[1], Ruyi Jin[1], Jiayu Liu[1], Ruilin Luo[1], Yinghan Zhang[1], Lin Zhu[#2], Xiaoli Peng*[1]

[1] College of Food Science and Engineering, Northwest A&F University, Yangling, Shaanxi, 712100, China

[2] Qinling National Botanical Garden, Xi'an, Shaanxi, 710061, China

*Corresponding author: Xiaoli Peng

Email addresses: pxlpxh@163.com

Fax: +86-29-87092817 a

ORCID: 0000-0003-3676-892X (Xiaoli Peng)

[#]Additional corresponding author: Lin Zhu

Email addresses: zhulinss@126.com

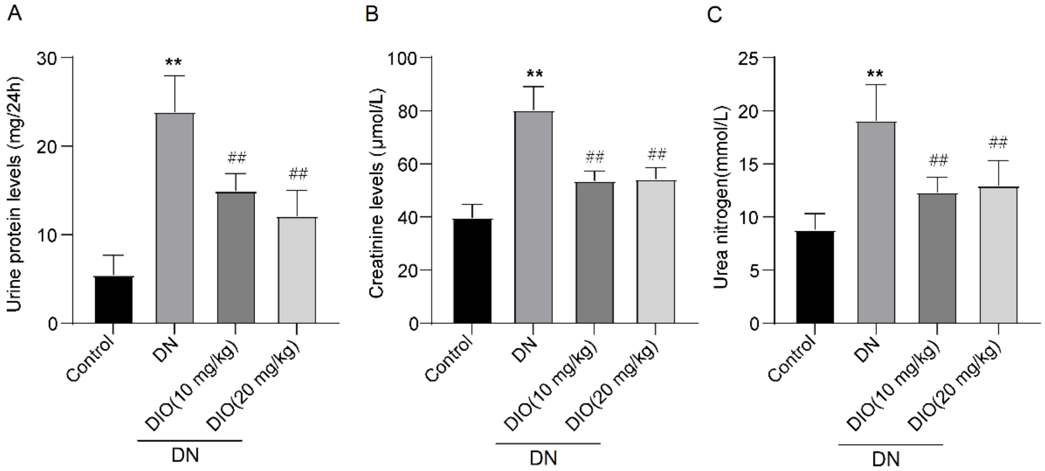

Figure S1. DIO reduced urine protein, serum urea nitrogen, and serum creatinine levels in DN rats. (A) The level of urine protein. (B) The level of creatinine. (C) The level of urea nitrogen. Data were presented as mean ± SD. $^{**}P < 0.01$ versus control group; $^{\#\#}P < 0.01$ versus DN group.

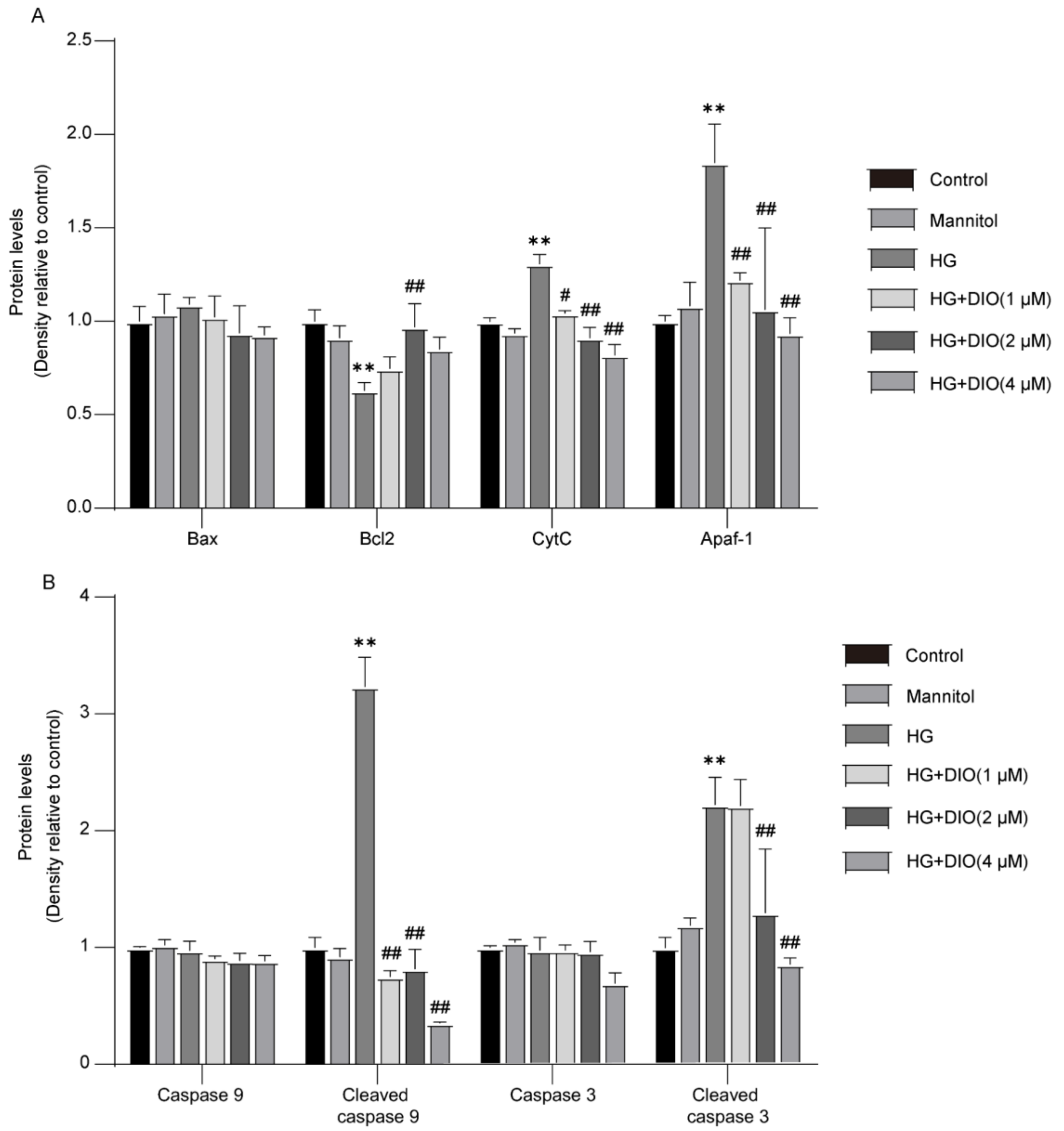

Figure S2. DIO ameliorated mitochondria-mediated apoptosis in HG-treated HK-2 cells. **(A)** Relative protein expressions of Bax, Bcl2, CytC, and Apaf-1 in HK-2 cells. **(B)** Relative protein expressions of caspase 9, cleaved caspase 9, caspase 3, and cleaved caspase 3 in HK-2 cells. Data were presented as mean ± SD. *$P < 0.05$, **$P < 0.01$ versus control group; $^{\#}P < 0.05$, $^{\#\#}P < 0.01$ versus HG group

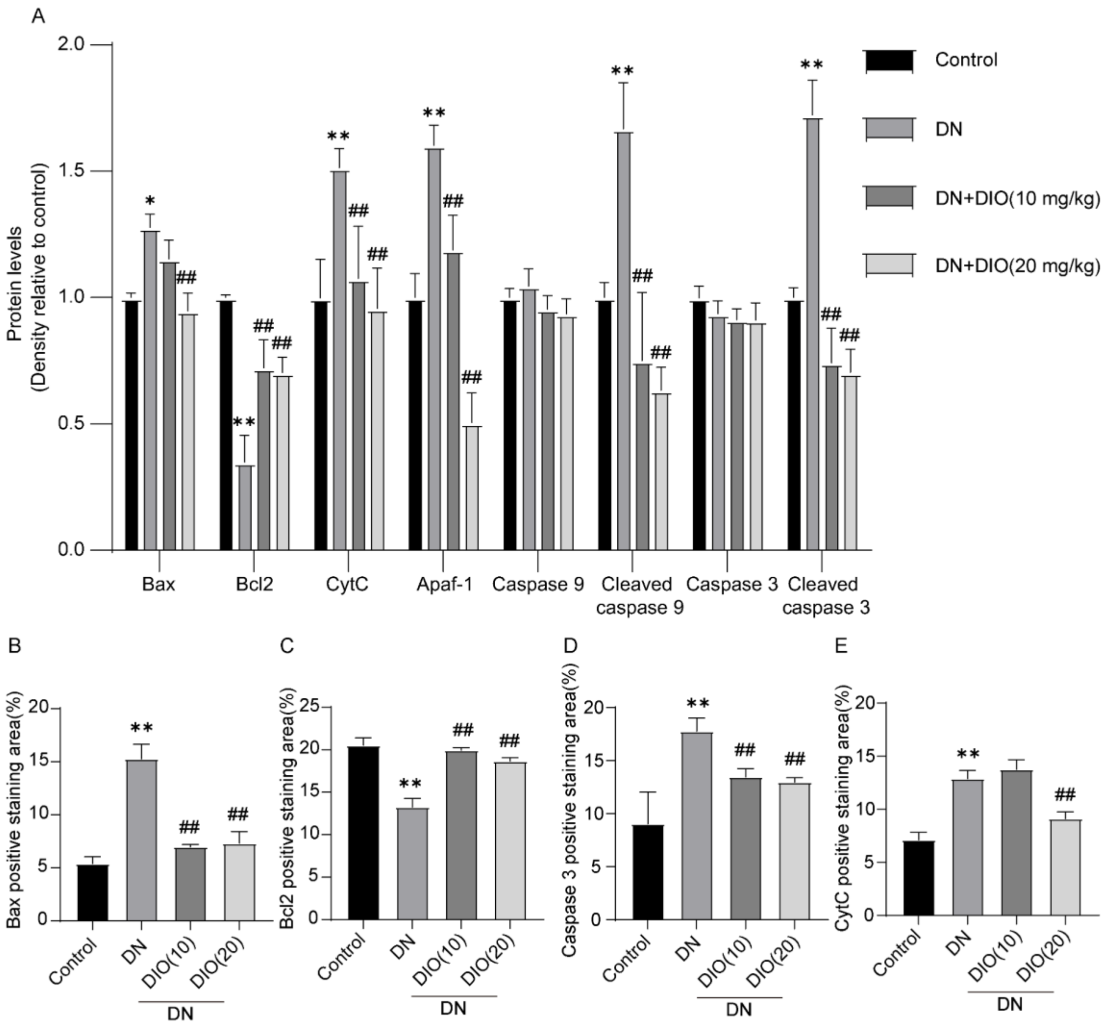

Figure S3. DIO ameliorated mitochondria-mediated apoptosis in the kidney of DN rats. **(A)** Relative protein expressions of Bax, Bcl2, CytC, Apaf-1, caspase 9, cleaved caspase 9, caspase 3, and cleaved caspase 3 in rat kidneys. **(B)** Bax positive staining area in immunohistochemistry. **(C)** Bcl2 positive staining area in immunohistochemistry. **(D)** Caspase 3 positive staining area in immunohistochemistry. **(E)** CytC positive staining area in immunohistochemistry. Data were presented as mean ± SD. *$P < 0.05$, **$P < 0.01$ versus control group; $^{\#}P < 0.05$, $^{\#\#}P < 0.01$ versus DN group.

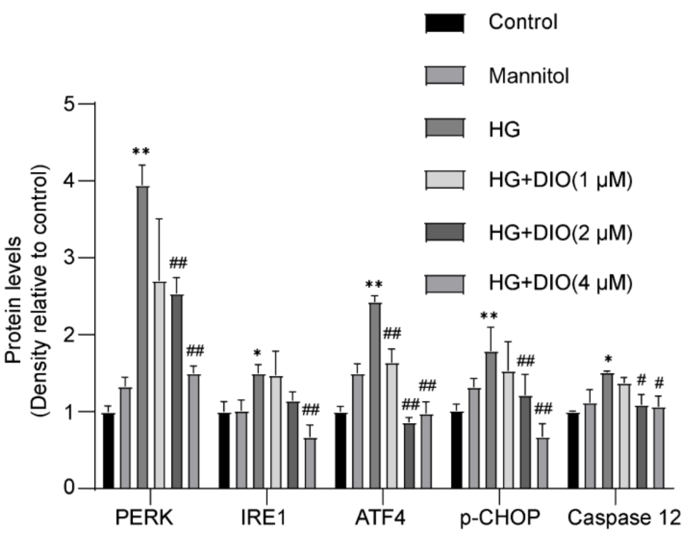

Figure S4. DIO ameliorated ER stress-mediated apoptosis in HK-2 cells. Relative protein expressions of PERK, IRE1, ATF4, p-CHOP, and Caspase 12. Data were presented as mean ± SD. $*P < 0.05$, $**P < 0.01$ versus control group; $^{\#}P < 0.05$, $^{\#\#}P < 0.01$ versus HG group.

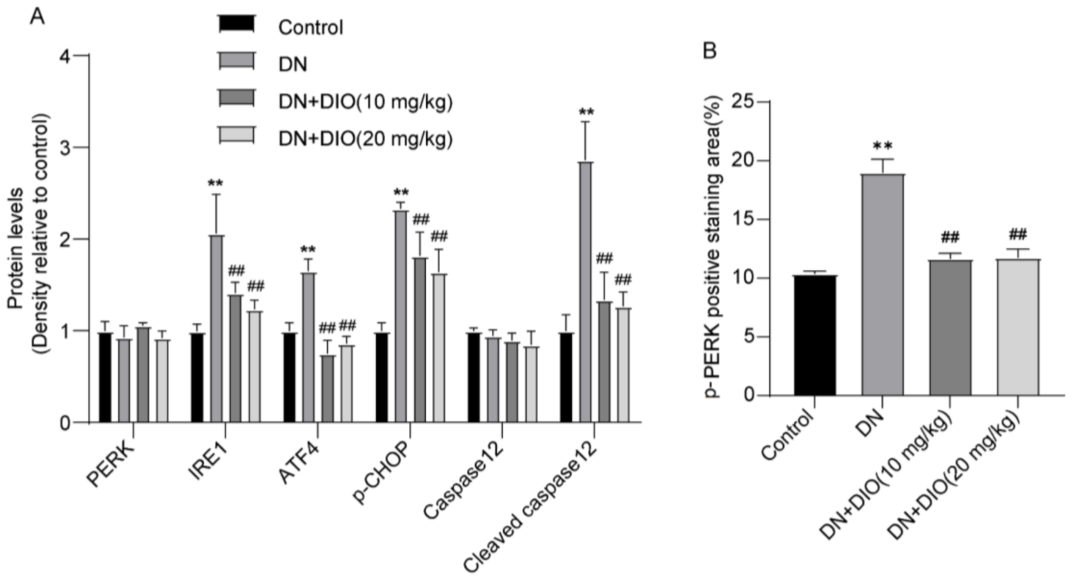

Figure S5. DIO ameliorated ER stress-mediated apoptosis in the kidneys of DN rats. **(A)** Relative protein expressions of PERK, IRE1, ATF4, p-CHOP, Caspase 12, and Cleaved caspase 12. **(B)** p-PERK positive staining area in immunohistochemistry. Data were presented as mean ± SD. **P* < 0.05, ***P* < 0.01 versus control group; $^{\#}P < 0.05$, $^{\#\#}P < 0.01$ versus DN group.

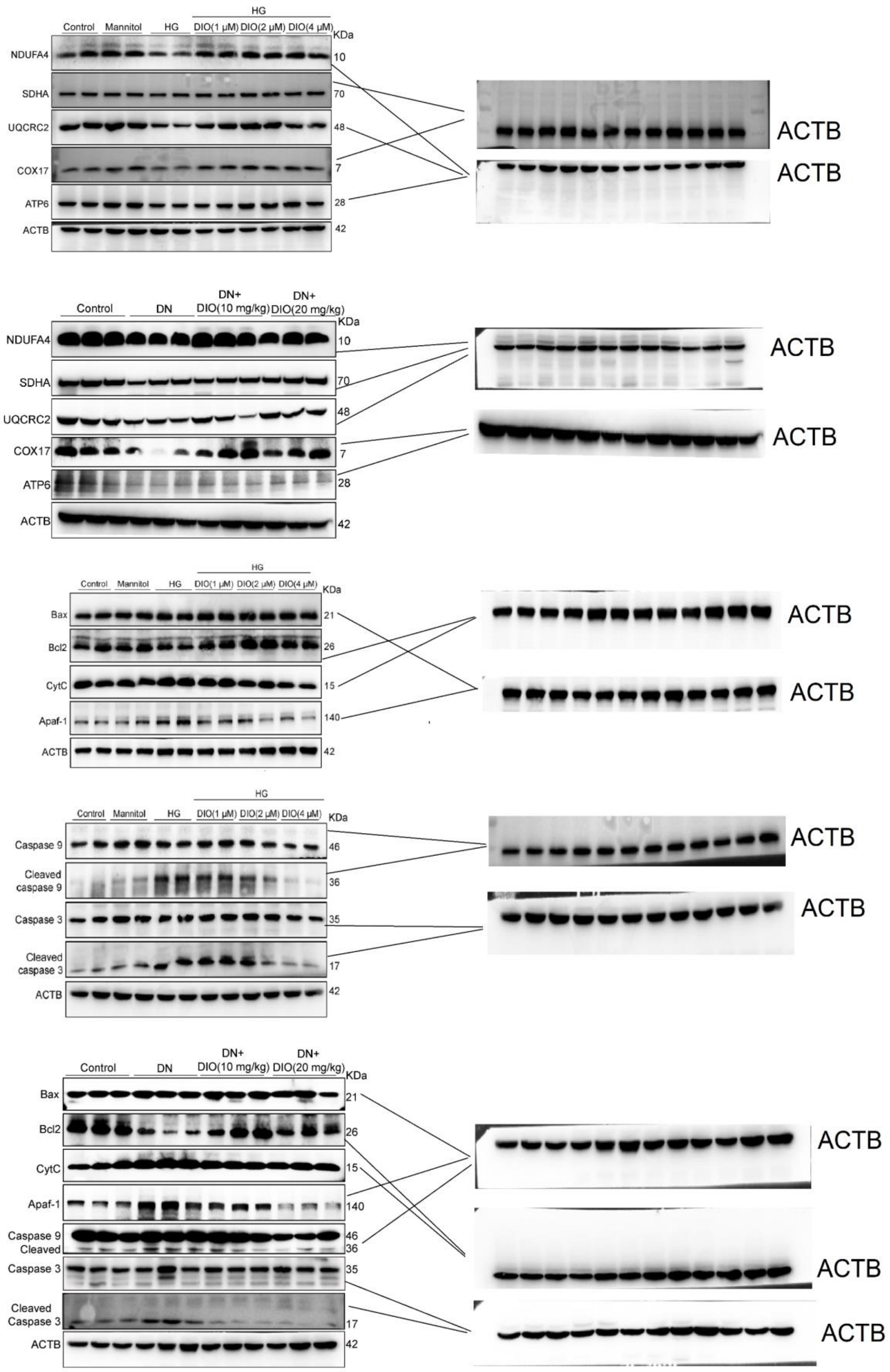

Figure S6. Western blot images and their corresponding internal controls.

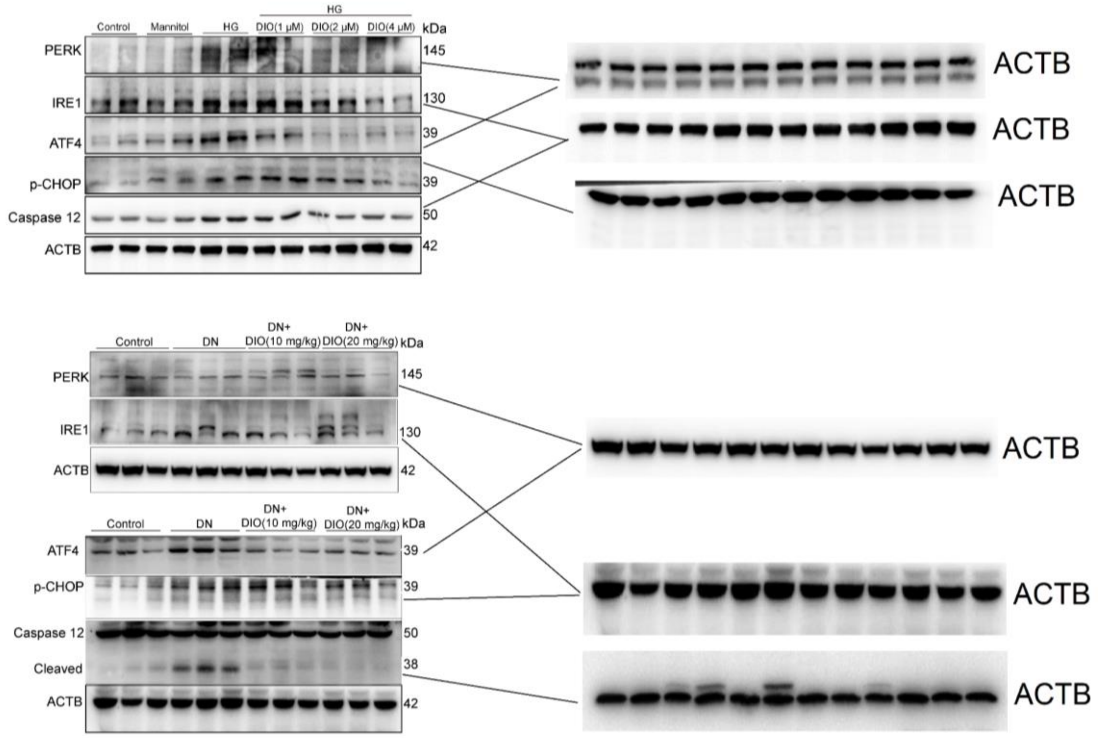

Figure S6 (continued). Western blot images and their corresponding internal controls.

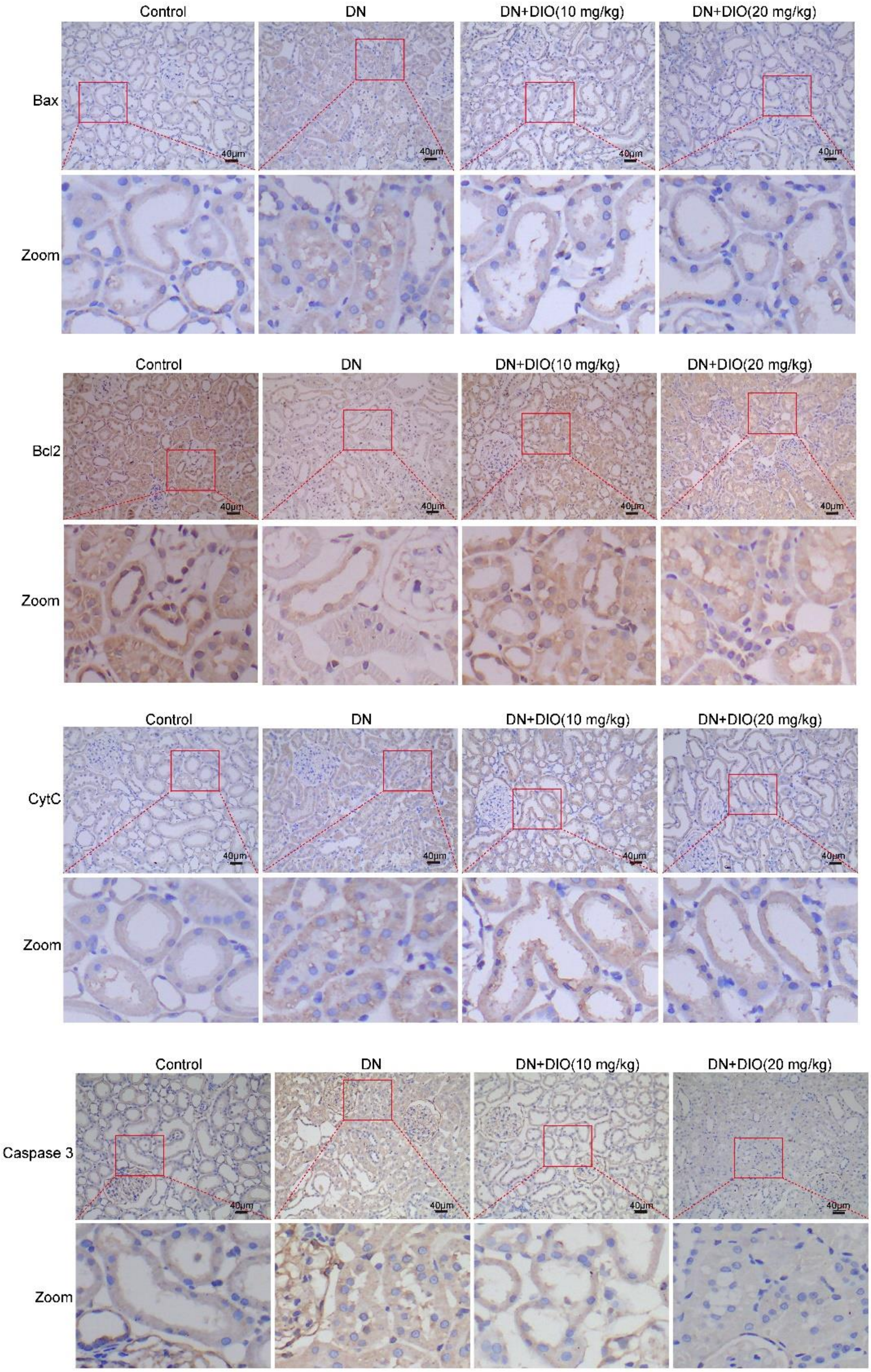

Figure S7. The representative immunohistochemistry images and their partial enlarged images of Bax.

Bcl2, CytC, Caspase 3, ATP8, and p-PERK.

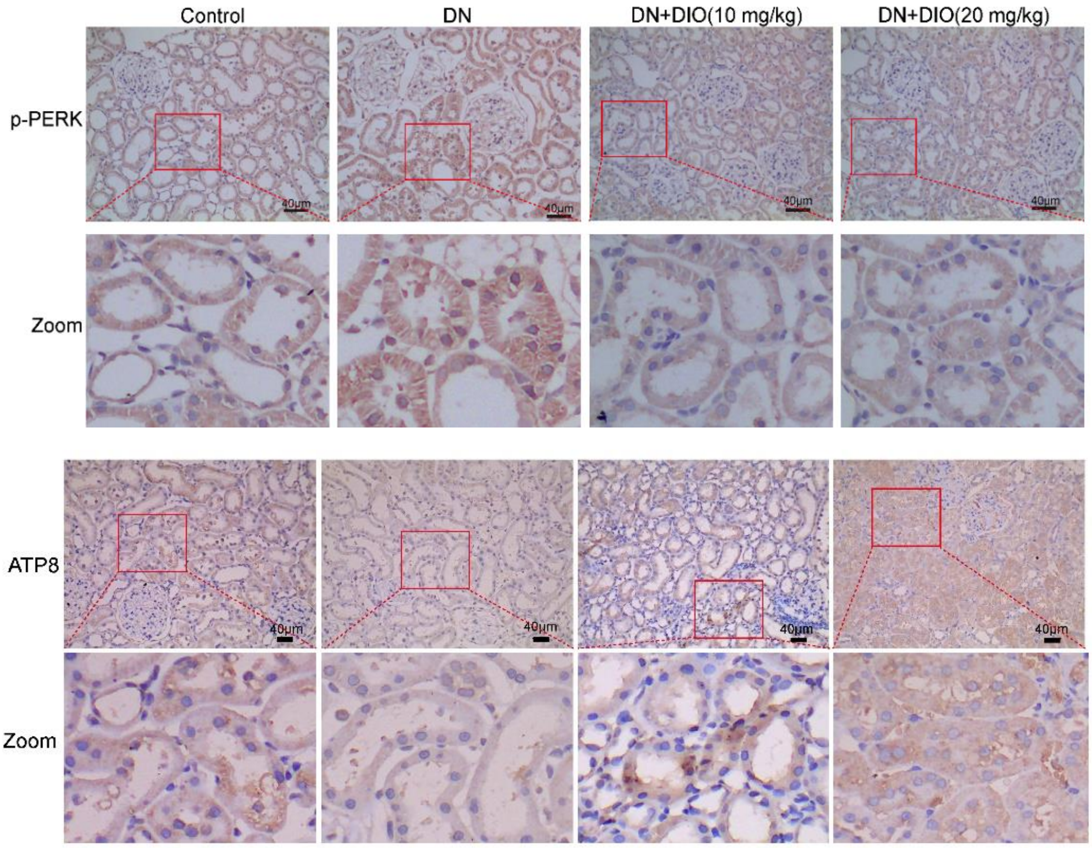

Figure S7 (continued). The representative immunohistochemistry images and their partial enlarged images of Bax. Bcl2, CytC, Caspase 3, ATP8, and p-PERK.
